# 

**Published:** 1890-01

**Authors:** 


JANUARY. 1890.

### THE AMERICAN JOURNAL OF DENTAL SCIENCE.

PUBLISHED MONTHLY.

$2.50 PER ANNUM.

EDITED BY

F. J. S. GORGAS, M. D., D. D. S.

Vol. 23. THIRD SERIES. No. 9.

BALTIMORE:

SNOWDEN & COWMAN, 9 W. FAYETTE STREET.

LONDON:
TRUBNER & CO., 60 PATERNOSTER ROW.

PAYABLE IN ADVANCE.

